# Quantitative detection of caffeine in beverages using flowing atmospheric-pressure afterglow (FAPA) ionization high-resolution mass spectrometry imaging and performance evaluation of different thin-layer chromatography plates as sample substrates

**DOI:** 10.1007/s00216-022-04045-z

**Published:** 2022-04-20

**Authors:** Maximilian Heide, Cristian C. Escobar-Carranza, Carsten Engelhard

**Affiliations:** 1 Department of Chemistry and Biology, University of Siegen, Adolf-Reichwein-Str. 2, 57076 Siegen, Germany; 2 Research Center of Micro- and Nanochemistry and (Bio)Technology, University of Siegen, Adolf-Reichwein-Str. 2, 57076 Siegen, Germany

**Keywords:** Ambient desorption/ionization, Mass spectrometry, Flowing atmospheric-pressure afterglow, Thin-layer chromatography, Quantification

## Abstract

Ambient desorption/ionization mass spectrometry (ADI-MS) is widely used as a rapid screening tool of samples in their native state without sample preparation. While analysis times are much less than 1 min per sample, one challenge of ADI-MS is the possibility to perform quantitative analysis of analytes in complex matrices. Typically, the goal is to probe a variety of different analytes in a complex matrix from a solid, liquid, or otherwise uncharacterized surface in the open air in front of the MS inlet. In this study, it is demonstrated that a carefully selected surface for analyte spot sampling and co-deposited isotopically labeled standards both significantly improve the capabilities of flowing atmospheric-pressure afterglow (FAPA) high-resolution (HR) MS for direct quantitative analysis. Specifically, a systematic study of different surfaces (glass, steel mesh, high-performance thin-layer chromatography (HPTLC) stationary phases including silica, reversed-phase (RP)-modified silica, and cyano (CN)-modified silica) and their suitability for spot sampling with FAPA-MS was performed. A set of different caffeine-containing standards and beverages (Red Bull, Coca-Cola, coffee, and black tea) was deposited on the surfaces and direct FAPA-HR-MS analysis of caffeine was performed using internal calibration with co-deposited ^13^C_3_-caffeine. For TLC surfaces, it was demonstrated that quantitative results could be achieved with the matrix and concomitants present and that a preceding chromatographic separation was not mandatory for this application. In addition, the use of a CN-HPTLC surface resulted in a significantly more intense caffeine signal in the beverage samples compared to the other surfaces studied, with the highest increase compared to the silica (200-fold higher) and the lowest increase compared to the steel mesh (30-fold higher). The utilization of TLC-based surfaces as sample carriers is considered an attractive tool in the ADI-MS toolbox for fast and efficient mass spectrometric investigations of complex samples without time-consuming sample preparation.

**Graphical Abstract:**

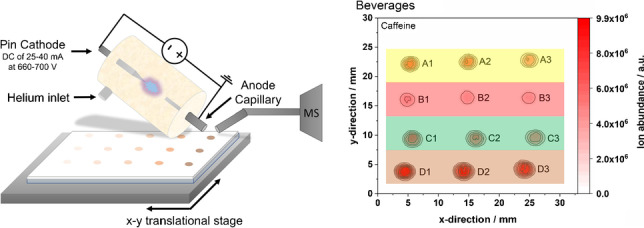

**Supplementary Information:**

The online version contains supplementary material available at 10.1007/s00216-022-04045-z.

## Introduction

Caffeine is the main active ingredient in coffee, tea, energy drinks, and other beverages. It is considered the most commonly used psychoactive substance worldwide because coffee and tea are the second most consumed beverages after water [1–3]. Caffeine can be found as a natural alkaloid in a variety of plants and its biosynthetic pathway has already been elucidated in detail [4, 5]. The IUPAC name 1,3,7-trimethylxanthine reveals that caffeine is structurally derived from xanthine, a purine base consisting of a pyrimidine ring connected to an imidazole ring [4].

Chemical analysis of coffee samples is typically carried out using high-performance liquid chromatography (HPLC) and ultra-high-performance liquid chromatography (UHPLC) systems with UV/VIS detection and reversed-phase columns [6–8]. Additionally, liquid chromatography–mass spectrometry (LC–MS) can be helpful when caffeine is present at lower concentrations or a complex matrix is involved. While LC–MS methods provide excellent limits of detection (LOD) in the low µg-per-liter-range, they often require preceding analyte extraction from coffee or tea samples, which, in total, can be time-consuming and costly. The food industry is, therefore, interested in direct analysis methods such as Fourier transform infrared spectroscopy (FTIR) followed by chemometric data analysis such as partial least square regression (PLS-R) and principal component analysis (PCA). For example, not only the detection of caffeine but also the discrimination between different coffee varieties, degree of roasting, and geographic origin of the plants was demonstrated using solvent extraction and FTIR [9–11]. The main drawback of FTIR-based methods is the comparably high LOD in the mg/L range and the need for time-consuming sample extraction to avoid matrix interferences with excipients [11]. Additionally, it is necessary to have access to and appropriate experience in chemometrics and multivariate data analysis to process the data.

Because the conventional methods such as FTIR and LC–MS are rather time-consuming, ambient desorption/ionization mass spectrometry (ADI-MS) is a promising candidate for a straightforward quantitative analysis approach, which also includes highly selective information when coupled to high-resolution mass spectrometers. The research field of ADI-MS started with the introduction of desorption electrospray ionization (DESI) by Cooks et al. and direct analysis in real-time (DART) by Cody et al. as new ionization sources for rapid analysis at ambient conditions [12, 13]. Till today, about 50 ADI sources that utilize laser-, plasma-, or spray-based desorption and ionization were developed for mass spectrometric applications [14]. One key aspect of all these sources is the possibility to probe samples in their original state without or with only minimal sample preparation. In laboratory practice, this would help to reduce or eliminate extraction protocols and chromatography-based separations prior to mass spectrometric analysis. On the other hand, this presents challenges to the mass spectrometer including higher loads of matrix and, in turn, might result in severe matrix effects, analyte ion suppression, and changes in ion transmission. Several studies that discuss these challenges in detail were summarized in a review published by Shelley and coauthors [14] in 2018. One approach to reduce ion suppression and matrix effects is to combine ADI sources with planar chromatography techniques such as thin-layer chromatography (TLC) or high-performance thin-layer chromatography (HPTLC). For example, the benefit of coupling TLC to DESI-MS [15–17], DART-MS [18–20], low-temperature plasma (LTP) [21], and molecular ionization-desorption analysis source (MIDAS) [22, 23] has been demonstrated in several studies. Other plasma-based ADI sources such as the flowing atmospheric-pressure afterglow (FAPA) source were used in TLC-ADI-MS studies as well [24, 25]. One advantage of plasma-based ADI sources compared to spray-based sources is the absence of additional solvent and solvent vapor, thus, minimizing analyte diffusion and broadening of the sample spot on the respective surface of interest. In addition, FAPA-MS experiments have the potential to be conducted with shorter analysis times and improved LODs while limiting the sample preparation effort. In an earlier publication from our group, the influence of different TLC parameters (thickness, particle size distribution, modification type) and drying conditions on analyte ion signals in mass spectrometric imaging with FAPA-MS [24] was studied. As a proof-of-principle example, caffeine in energy drinks was quantified using normal-phase (NP)-HTPLC-FAPA-MS and isotope dilution analysis (IDA) [24].

In this study, the influence of different sampling surfaces on the analyte signal response in FAPA-HR-MS and the possibility of accurate quantification based on isotopically labeled standards were systematically investigated and extended to other samples. For the TLC surfaces, it was also compared whether a prior planar chromatography step is important for both best analyte signal response and accurate quantification in the presence of a complex matrix and various concomitants. In addition, it was investigated whether matrix effects would differ for various sampling surfaces and lead to significant matrix-induced suppression of the analyte ion signal in direct FAPA-HR-MS analysis of beverage samples on TLC plates. For this purpose, pure caffeine standards with different concentrations were also investigated comparatively. In another experiment, FAPA-MS was used in transmission mode to perform quantitative analysis using the isotope dilution approach and results were compared to scanning FAPA-MS. The obtained quantitative results for the beverage samples were compared and validated by HPLC-UV measurements.

## Materials and methods

### Reagents

Propan-2-ol (analytical grade) and *n*-heptane (analytical grade) were purchased from VWR Chemicals (Radnor, PA, USA) and methanol (HPLC grade) was purchased from Fisher Chemicals (Hampton, NH, USA). Bidistilled water was freshly produced with a distillation apparatus from Heraeus-Quarzschmelze GmbH (Hanau, Germany) in the laboratory. Caffeine (> 99.9% chemical purity) and ^13^C_3_-caffeine standard solution (1 mg/mL in methanol, 99 atom % ^13^C, > 99% chemical purity) were obtained from Merck KGaA (Darmstadt, Germany). The used HPTLC plates including C18 reversed-phase (RP)-HPTLC LiChrospher®, NP-HPTLC, and cyano (CN)-modified HPTLC were all provided by Merck KGaA (Darmstadt, Germany). Microscope slides were acquired from Carl Roth GmbH + Co. KG (Karlsruhe, Germany), and uncoated TLC glasses were used from MACHEREY–NAGEL (Düren, Germany). Red Bull, Coca-Cola, coffee, and black tea as caffeine-containing beverages were obtained from local grocery stores.

### Preparation of caffeine standard solutions

A caffeine stock solution was prepared by dissolving caffeine (20.4 mg) in methanol (20.0 mL) to give a concentration of 1.02 mg/mL. Based on the stock solution, a dilution series was produced yielding concentrations of 20.4, 10.2, 2.04, and 1.02 μg/mL. ^13^C_3_-caffeine was added to all solutions of the series to give internal standard concentrations of 5.0 μg/mL each. For the pure caffeine studies, a methanolic stock solution (498 μg/mL) was prepared and diluted in tenfold dilution steps to finally achieve the lowest concentration of 49.8 ng/mL.

### Preparation of beverage samples and sample extracts

Beverage samples were used without sample preparation for direct analysis by FAPA-MS. In addition, liquid–liquid extraction of the beverages was performed as a comparison and for validation by HPLC–UV experiments. Coca-Cola and Red Bull were degassed by ultrasonification for 10 min. Tea (1.019 g) and coffee (3.077 g) were mixed separately with 50 mL of water and heated till boiling. The suspensions were boiled for 4 min in a covered beaker and then filtered (Whatman® Schleicher & Schuell filter papers, grade 595 ½, Ø 240 mm) to give the beverages for further experiments. One aliquot was set aside for direct analysis experiments and another aliquot was further extracted.

For liquid–liquid extraction, the respective beverage samples (5 mL) were transferred into a separation funnel and bidistilled water (10 mL) and aqueous sodium carbonate solution (20 w%, 1 mL) were added. The aqueous phase was extracted with chloroform (3 × 20 mL) and the combined organic phases were further evaporated to dryness using a TurboVap II (Biotage, Uppsala, Sweden). During the drying process, the walls of the evaporation containers were rinsed three times with methanol (10, 5, and 3 mL respectively) to assure the highest recovery of analyte in the extracts. The samples were reconstituted in methanol and transferred to 5-mL volumetric flasks, which were filled to the mark with methanol to a total volume of 5 mL. The solutions were ultrasonicated and transferred to 1-mL vials after filtration with 0.45-µm syringe filters for further analysis.

For quantification of caffeine in the beverages via isotope dilution analysis, an aliquot of either 80 or 75 μL of beverage was mixed with either 20 or 25 μL of methanolic ^13^C_3_-caffeine solution (1 mg/mL) to give a total volume of 100 μL. To ensure complete homogenization, the mixture was vortexed for 2 min.

The prepared beverage samples and all ^13^C_3_-caffeine-containing solutions were stored at − 30 °C. The extracts were stored at 4 °C.

### HPTLC preparation

For the HPTLC experiments, 4 cm × 5 cm plates were prepared. This was done for all used HPTLC types to guarantee reproducibility and comparability. For the separation, a small volume (1 μL, if not stated otherwise) of the respective sample, either untreated or extract-based, was deposited with a 1-μL capillary onto the plate. Typically, 4 spots of an individual sample were applied to a plate to generate a useful data set. The spots were dried on a hot plate at 50 °C for at least 5 min to ensure complete solvent evaporation. Separation of the analyte mixtures was performed in a MINIPLAK glass developing chamber (Fungilab Inc., New York, NY, USA) using a mobile phase mixture of propan-2-ol, *n*-heptane, and water (7:3:1 (*v/v/v*)), which achieved separation of matrix compounds from the caffeine and structurally related compounds (data not shown). Depending on the plate type, the separation took between 12 and 25 min. After separation, the plates were dried at 50 °C on a hot plate for 15 min and investigated in a UV cabinet (UV-Cabinet II, CAMAG, Muttenz, Switzerland). For further mass spectrometric analysis, the plates were placed in front of the mass spectrometer and the respective ADI source.

### Glass surfaces and mesh preparation

To investigate the necessity of planar chromatography for reliable quantification, additional experiments were performed using microscope slides and uncoated TLC glass plates made of soda-lime glass as sample carrying surfaces for mass spectrometric imaging and analysis. The microscope slides with original dimensions of 76 mm × 26 mm and a thickness of 1 mm were bisected for the experiments to give pieces of 38 mm × 26 mm dimensions and to fit them into the TLC plate holder. For analyses, three droplets (1 μL each) of sample solution were deposited on the respective glass surface. Due to the lack of a backing material, these droplets became indistinct and blurred out compared to the narrow stains achievable on HPTLC plates. This also limited the number of droplets on the glass sides to avoid signal overlapping due to indistinct regions on the respective plates. Glass slides were extensively cleaned with methanol before the measurement and no reuse was intended. Uncoated TLC glass plates were cut into the same dimensions as the HPTLC plates and treated similarly to the microscope slides. However, sample droplet application was found to be easier on the respective plate surface.

For analysis in transmission mode (TM mode), stainless-steel meshes were used (austenitic corrosion-resistant stainless-steel wire mesh 1.4301, mesh size 315 µm, wire cross sect. 200 µm). For fixation of the meshes, an aluminum rail was positioned in front of the mass spectrometer inlet. The rail was mounted with an aluminum pinhole card in which the meshes were inserted. For mass spectrometric analysis, the clean meshes were loaded with sample solution (1 μL per spot), which was allowed to dry for 5 min at room temperature to decrease solvent signals in the spectra.

### Instrumentation

An Exactive Orbitrap (Thermo Fisher Scientific, Bremen, Germany) was used for high-resolution MS analysis. The instrument was calibrated with an ESI–MS method daily before the respective experiments to achieve high resolution and mass accuracy. For mass calibration, the Pierce LTQ ESI Positive Ion Calibration Solution (Thermo Fisher Scientific, Bremen, Germany) consisting of caffeine (20 µg/mL), Met-Arg-Phe-Ala (MRFA, 1 µg/mL), and Ultramark 1621 (0.001%) in an aqueous solution of acetonitrile (50%), methanol (25%), and acetic acid (1%) was used, which covers a mass-to-charge ratio (*m/z*) range of 138 to 1922 m*/z*. The resolution during the experiments was set to 50,000 (at 200 m*/z*) and the maximum injection time was set to 10 ms. The maximum day-to-day mass measurement uncertainty was < 5 ppm (cf. Table 1).

**Table 1:** Ion traces (XICs) for mass spectrometric imaging and quantification

Analyte	Ion trace for XIC	Measured *m/z*	Species	Rel. mass accuracy^1^
Caffeine	195.0855–195.0886	195.0868	[**M**^**1**^ + H]^+^	< 4 ppm
^13^C-labeled caffeine	198.0952–198.0984	198.0968	[**M**^**2**^ + H]^+^	< 4 ppm
Caffeine fragment	138.0644–138.0666	138.0655	[**M**^**1**^-C_2_H_3_NO + H]^+^	< 4 ppm
^13^C-labeled caffeine fragment	140.0710–140.0732	140.0722	[**M**^**2**^-C^13^CH_3_NO + H]^+^	< 5 ppm

^1^Based on bias to predicted exact mass of the respective ion calculated in Xcalibur. **M**^**1**^ represents caffeine with natural isotope abundance and **M**^**2**^ represents isotope-labeled ^13^C_3_-caffeine

**Fig. 1:**
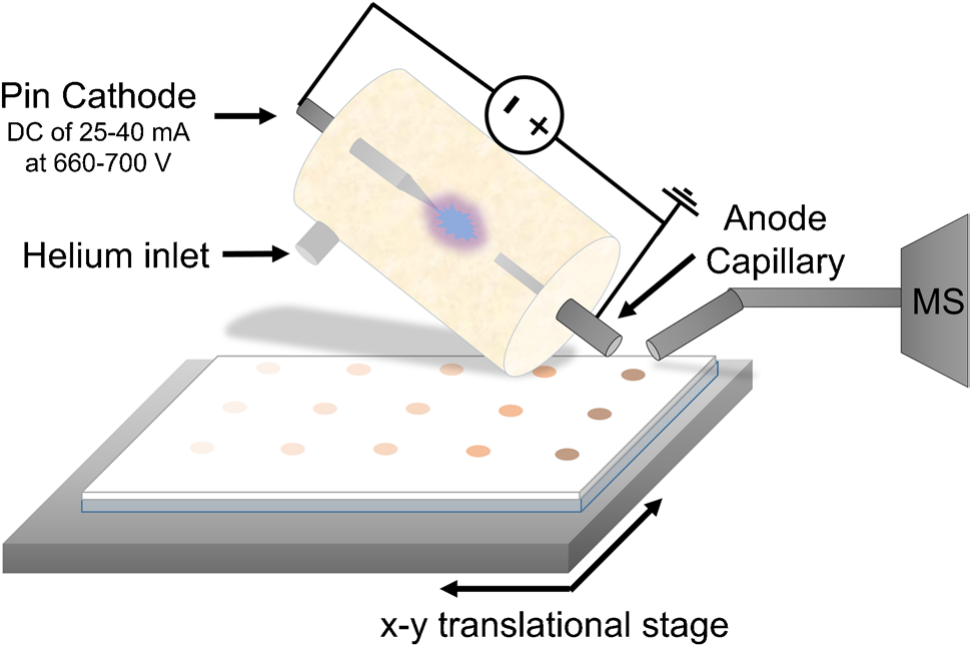
Schematic setup of the pin-to-capillary FAPA source for surface analysis of TLC plates. The FAPA source figure was adapted from Shelley et al. [26]

For all ADI-MS experiments, a home-built pin-to-capillary FAPA source was used as described by Shelley and coauthors [26] and as schematically depicted in Fig. 1.

A Macor® ceramic (Schröder Spezialglas, Ellerau, Germany) was used as the discharge chamber. A sharpened stainless-steel electrode (1.6 mm outer diameter (o.d.)) was positioned on one side of the chamber and a capillary electrode (1.6 mm o.d., 1.0 mm inner diameter (i.d.), 28 mm length) was positioned on the other side of the chamber. The distance between pin and capillary electrodes was kept at 8 mm during the experiments. To operate the discharge, a negative potential was applied to the pin cathode through a 5 kΩ resistor in a current-controlled mode using a DC power generator (Kepco, Flushing, NY, USA) with currents ranging from 25 to 40 mA and resulting potentials ranging from 660 to 700 V. Grade 5 Helium (Messer Industriegase GmbH, Siegen, Germany) was used as the discharge gas at a flow rate of 750 mL/min if not stated otherwise. No other grid electrodes (cf. DART [27]) were used. The helium flux was reproducibly adjusted by an EL-FLOW® Select mass flow controller (Bronkhorst Deutschland Nord GmbH, Kamen, Germany).

The ADI source was mounted on an aluminum clamping plate surrounded by an acrylic glass housing to ensure isolation from interferences in the laboratory. The clamping plate includes a mechanic device with similar fittings as the conventional ESI source to position the ADI source reproducibly and stable in front of the instrument.

For TM mode experiments, the conventional inlet capillary was used and an aluminum pinhole card was screwed onto the clamping plate. The FAPA source was operated at an angle of 0° towards the sample holding mesh. The distance between the FAPA outlet and the MS inlet capillary was kept at 16.5 mm and the distance between the mesh and the MS inlet capillary was kept at approx. 1 mm. To improve reproducibility for the TM mode experiments, the data acquisition was started after the total ion current (TIC) variation decreased below 10% and data were then recorded for 15 s.

For HPTLC and glass slide experiments, the conventional capillary was replaced by a curved inlet capillary with an i.d. of 0.6 mm and about 4 cm extension. Also, the mesh holding system was replaced by a HPTLC plate and microscope slide holding system with a motorized x–y stage (Newport Corporation, Irvine, CA, USA) controlled by a LabVIEW-based (Version 11.0, 2011, National Instruments, Austin, TX, USA) software. The angle between the FAPA outlet and the plate surface was adjusted to 60°. The distance between the FAPA outlet and MS inlet capillary was kept between 1.5 and 2.0 mm, whereas the distance between the FAPA outlet and plate surface was kept below 1 mm, and the distance between plate surface and MS inlet capillary was kept around 0.5 mm. The HPTLC plates were locomoted beneath the FAPA outlet and MS inlet with the motorized x–y-stage for mass spectrometric imaging. Line scans were performed at a speed of 0.3 mm/s in x-direction followed by a line-to-line step with a distance of 0.5 mm in the y-direction to cover the region of interest (ROI) based on the spatial resolution of the desorption spot. Data acquisition was triggered for each line by the control software.

### Molecular mapping and data analysis

The mass spectrometric data from TM mode and surface mapping experiments were collected with the Exactive Tune software (11.0 SP3, Thermo Scientific, Bremen, Germany). Extracted ion chronograms (XICs) were used based on a mass accuracy range of ± 8 ppm for the respective ion of interest. The used ion traces are listed in Table 1. For further data handling and visualization, the XICs were transferred to Origin 2017 (OriginLab Corporation, Northampton, MA, USA). For TM mode analysis, the generated XICs intensities were used directly, and for the generation of molecular maps, the respective lines of each surface measurement were added up. The time on the x-axis was transferred to a distance based on the scan rate of 0.3 mm/s and the total number of lines defined the distance in the y-direction based on 0.5 mm steps between each line. With the additional information on the XICs intensities, 2D contour plot diagrams were generated. For quantitative analysis, the obtained areas of interest in the contour plots were integrated or the local maxima of the specific area were used. It was found that resulting concentrations did not deviate from each other significantly and only the standard deviation slightly increased when using the maxima. Thus, only the results based on the integrated ROI are presented throughout the manuscript if not stated otherwise.

Besides classic quantification based on external and internal standard calibration, which was performed for selected TM mode experiments, quantification using the isotope dilution analysis approach was performed. Details on this are provided in the supporting information.

### Safety considerations

The experimental setup used for the experiments includes high voltages and currents to power the FAPA source. All connections between the power supply and ADI source have to be insulated to prevent electric shock. A housing connected to the laboratory air ventilation that covers the FAPA source should be used to collect evaporated solvents and potentially toxic or corrosive chemicals and byproducts. During a running experiment with the FAPA source, all laboratory users should be informed and hazards should be pointed out to avoid accidents or safety risks.

## Results and discussion

### Comparison of different HPTLC stationary phases for quantification of caffeine in energy drinks using TLC-FAPA-MS

In earlier work from our group, promising results were obtained for direct caffeine analysis in energy drinks using FAPA-MS in combination with NP-HPTLC plates (24). Caffeine in three different energy drinks was separated from matrix compounds using planar chromatography before being probed by FAPA-MS. Quantification was achieved using isotopically labeled ^13^C_3_-caffeine and a preceding separation step using NP-HPTLC plates helped to adequately separate an isobaric interference (peak at *m/z* 198.0987; which supposedly is a water adduct ion of matrix compound *m/z* 180.0881, see ESM Fig. S4 in ref. [21]; it might also be considered that *m/z* 180.0881 could be the result of a water loss of *m/z* 198.0987) to the caffeine-^13^C_3_ standard ([M^2^ + H]^+^, *m/z* 198.0981). While stationary phase chemistries were investigated with neat standards in the same study, other HPTLC functionalities were not used for quantitative FAPA-MS analysis of samples with complex matrices. Therefore, in the first part of the present study, an investigation into the applicability of other HPTLC functionalities and beverage samples was carried out, also to check whether or not a similar isobaric interference would be observed. If that were to be the case, planar chromatography (or mass spectrometers with a higher mass resolving power) could become important for accurate results in this type of application.

**Fig. 2:**
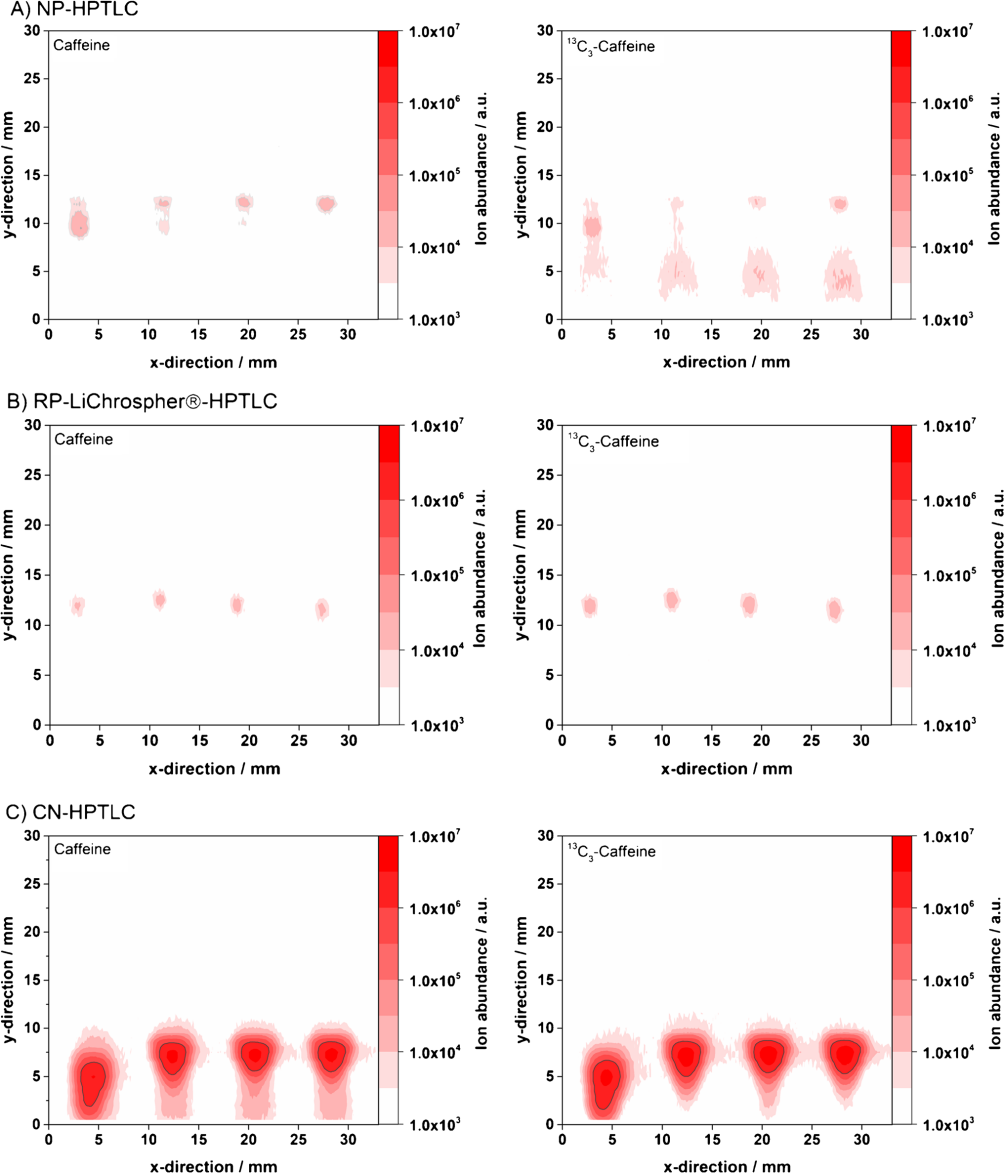
Energy drink loaded onto different HPTLC plates and analyzed by FAPA-MS imaging: mass spectrometric images of A caffeine (left) and ^13^C_3_-caffeine (right) on NP-HPTLC; B caffeine (left) and ^13^C_3_-caffeine (right) on RP-LiChrospher®-HPTLC; C caffeine (left) and ^13^C_3_-caffeine (right) on CN-HPTLC. The FAPA source was operated at a helium flow rate of 750 mL/min and a discharge current of 35 mA. The deposited sample volume was 1 µL per spot (320 ng analyte/spot). For caffeine and ^13^C_3_-caffeine, the mass traces for the [M + H]^+^-species were used given in Table 1

First, energy drinks (spiked with an internal ^13^C_3_-caffeine standard) were spotted onto different HPTLC plates (NP-, RP-LiChrospher®-, and CN-HPTLC), developed within 12–25 min (see “Materials and methods” for details), and probed by FAPA-MS in scanning mode. In Fig. 2, the influence of the HPTLC material on the mass spectral images of the caffeine analyte and the internal ^13^C_3_-caffeine standard is shown. Signal intensities for NP- and RP-LiChrosphere®-plates are similar in magnitude, whereas CN-plates show a 100-fold increase in signal intensities. While the isobaric interference mentioned above was observed here as well when using NP-HPTLC plates (Fig. 2A, ^13^C_3_-caffeine graph, two spots are visible in y-direction at 2–8 mm and at 10–12 mm), it was not observed with RP-LiChrospher®- or CN-HPTLC plates.

The reason could be that either (i) the isobaric interference is only present in the NP-HPTLC experiment or (ii) the species is chromatographically resolved from ^13^C_3_-caffeine only with the normal-phase but coelutes in the case of RP-LiChrospher®-, and CN-HPTLC. Here, a closer look into the calculated caffeine concentrations might help. Table 2 shows the calculated caffeine concentrations based on internal standardization with ^13^C_3_-caffeine. The presence of a coeluting isobaric interference in Fig. 2B, C would decrease the measured ratio R[^12^C/^13^C] based on Eq. S1. As a result, it would lead to a distinctive negative bias of the caffeine concentration (as described by Kuhlmann et al. [24] earlier in detail). However, as this is not observed in Table 2, it is assumed here that no or no significant contribution of an isobaric interference is present when RP-LiChrospher® and CN-HPTLC plates are used in this type of application. In conclusion of the first part, it was found that CN- and RP-LiChrospher®-HPTLC plates are attractive alternatives to NP-HPTLC plates. They give reliable and better results (improved RSDs and smaller bias, see Table 2) that are in good agreement with HPLC and manufacturer values. As a result, only these two types of materials were selected for the following experiments below.

**Table 2:** Quantitative analysis of caffeine in four dried energy drink sample spots on HTPLC plates using FAPA-MS and internal standardization. The results are based on the integrated ROI of the respective analyte ions present in the contour plots. A ratio of 20 µL internal standard and 80 µL sample was used and a volume of 1 µL per spot was applied. A fourfold determination was performed for each plate type as visualized in Fig. 2

HPTLC plate type	Caffeine concentration (mg/mL)	RSD	Bias to references^1^
NP	0.341 ± 0.028	8%	+ 5% (HPLC^2^) + 7% (manufacturer^3^)
RP-LiChrospher®	0.303 ± 0.010	3%	− 6% (HPLC^2^) − 5% (manufacturer^3^)
CN	0.337 ± 0.004	1%	+ 4% (HPLC^2^) + 5% (manufacturer^3^)

^1^Bias based on HPLC–UV reference experiments and manufacturer information on the caffeine content

^2^Measured caffeine concentration by HPLC–UV was 0.324 ± 0.006 mg/mL

^3^Caffeine concentration given by the manufacturer is 0.320 mg/mL

### Caffeine quantification in beverages and extracts on RP-LiChrospher®- and CN-HPTLC plates using planar chromatography vs. direct scanning FAPA-MS

**Fig. 3:**
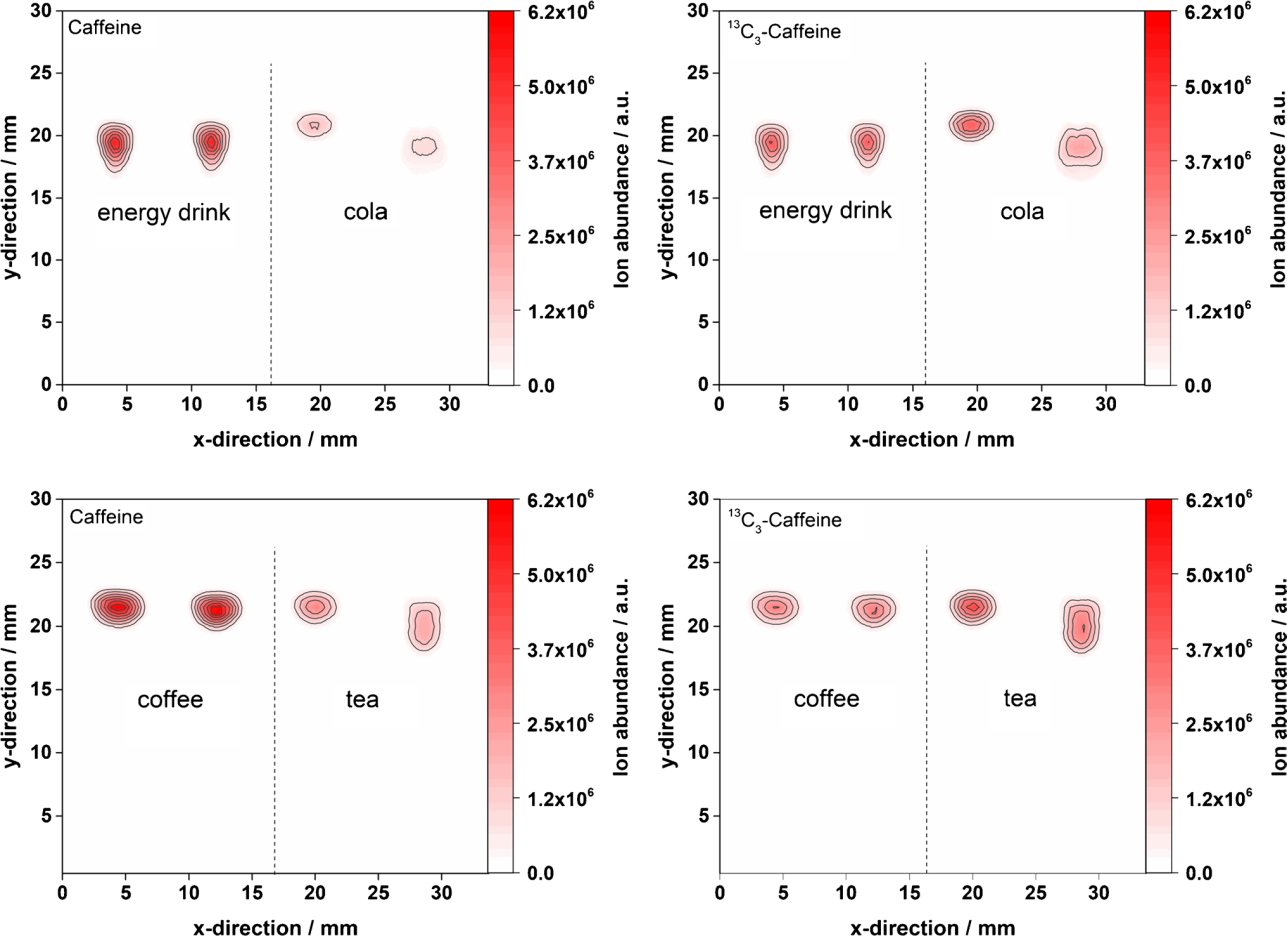
CN-HPTLC run of different beverages followed by FAPA-MS imaging: mass spectrometric images of caffeine and ^13^C_3_-caffeine on CN-HPTLC plates after separation from the matrix (not visualized) in an energy drink, cola, coffee, and tea, respectively. The FAPA source was operated at a helium flow rate of 750 mL/min and a discharge current of 35 mA. The deposited sample volume was 1 µL per spot (320 ng analyte/spot for energy drink, 97 ng analyte/spot for cola, 341 ng analyte/spot for tea, and 778 ng analyte/spot for coffee). For caffeine and ^13^C_3_-caffeine, mass traces for the [M + H]^+^-species are listed in Table 1

Because of the promising quantitative HPTLC-FAPA-MS results above, the study was expanded to other beverages that contain caffeine. In addition to energy drinks, coffee, tea, and cola were studied. Analytes were separated from the matrix on CN-HPTLC plates followed by FAPA-MS analysis. Quantitative results were obtained and the corresponding mass spectrometric images for caffeine in different beverages are depicted in Fig. 3. The highest caffeine concentrations were found in coffee followed by the energy drink and tea (similar content) and cola with the lowest caffeine concentration (cf. Table 3). These results were found to be in good agreement with HPLC–UV experiments, which were performed to validate the CN-HPTLC-FAPA-MS data. CN-HPTLC-FAPA-MS results for all beverages are summarized in Table 3 and compared to HPLC–UV data. Because a reproducible desorption/ionization cannot be taken for granted in ADI-MS [14, 27, 28], the results are considered very promising. Even though only a twofold determination was performed, very low standard deviations were achieved, which is remarkable in ADI-MS and indicates a high precision of the method. Unfortunately, the caffeine concentrations were found to be slightly off compared to HPLC–UV results especially for tea, which shows a negative bias on the order of 30% (based on [M + H]^+^ *m/z* 195.0868 and *m/z* 198.0968, concentration values indicated using the superscript letter “2” in Table 3). Additional investigations based on the fragments of caffeine and ^13^C_3_-caffeine (concentration values indicated using the superscript letter “3” in Table 3) gave better results for coffee but did not improve the results for the other samples. For example, the result for the energy drink was significantly biased compared to caffeine concentrations calculated using [M + H]^+^ ions. Clearly, the use of fragment ions was found to be not helpful in this type of matrix and shows that such strategies have to be evaluated individually based on the type of sample and matrix.

**Table 3:** Caffeine concentrations in different beverages determined after a CN-HPTLC run and FAPA-MS imaging. The results are based on the integrated regions of interest of the respective analyte ions present in the contour plots in Fig. 3. For energy drink and cola, 20 µL of standard and 80 µL of sample were mixed. For coffee and tea, 25 µL of standard and 75 µL of sample were mixed. For each beverage, analysis was performed twice

Beverage	Caffeine concentration (mg/mL)	RSD	Bias to reference^1^
Energy drink	0.340 ± 0.0001^2^	≤ 1‰^2^	+ 5%^2^
	0.404 ± 0.058^3^	14%^3^	+ 25%^3^
Cola	0.104 ± 0.0005^2^	≤ 1%^2^	+ 7%^2^
	–	–	–
Coffee	0.651 ± 0.0001^2^	≤ 1‰^2^	− 16%^2^
	0.699 ± 0.0005^3^	≤ 1‰^3^	− 10%^3^
Tea	0.222 ± 0.0005^2^	≤ 1%^2^	− 35%^2^
	0.232 ± 0.0002^3^	≤ 1‰^3^	− 32%^3^

^1^Caffeine concentration determined by HPLC–UV: energy drink: 0.324 mg/mL, cola: 0.097 mg/mL, tea: 0.341 mg/mL, coffee: 0.778 mg/mL, RSD ≤ 2% for all HPLC–UV measurements

^2^Results based on protonated molecular ions *m/z* 195.0868 and *m/z* 198.0968

^3^Results based on fragment ions *m/z* 138.0655 and *m/z* 140.0722

**Fig. 4:**
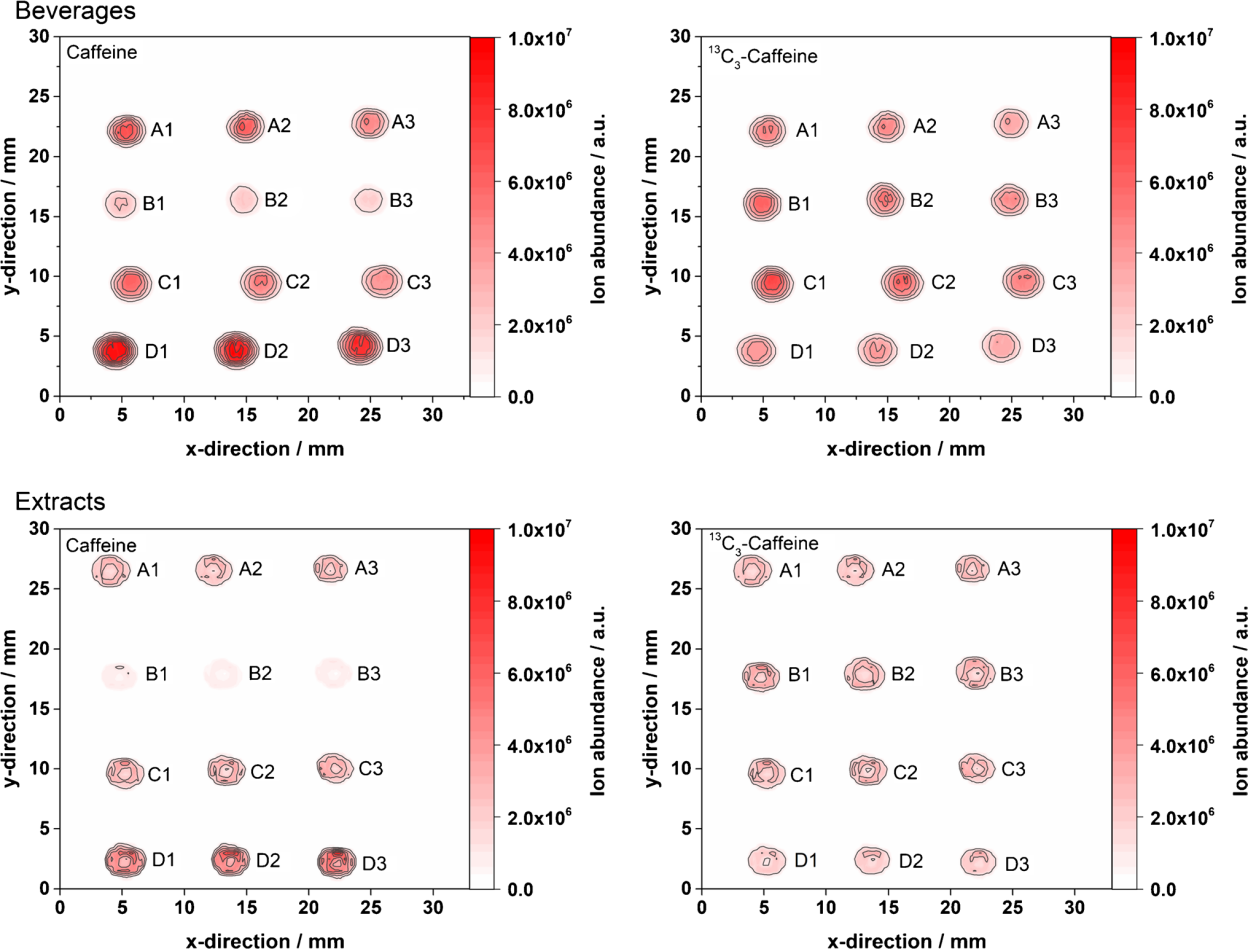
Direct FAPA-MS analysis of dried spots on CN-HPTLC plates: mass spectrometric images of caffeine (left) and ^13^C_3_-caffeine (right) in beverages (upper part) and beverage extracts (lower part). Samples included an energy drink (A1–A3), cola (B1–B3), tea (C1–C3), and coffee (D1–D3). The FAPA source was operated at a helium flow rate of 750 mL/min and a discharge current of 35 mA. The deposited sample volume was 1 µL per spot (320 ng analyte/spot for energy drink, 97 ng analyte/spot for cola, 341 ng analyte/spot for tea, and 778 ng analyte/spot for coffee). For caffeine and ^13^C_3_-caffeine, mass traces for the [M + H]^+^-species are listed in Table 1

To investigate potential matrix effects on mass spectrometric imaging and the quantitation approach, the original beverage samples as well as liquid–liquid extracts of the four beverages (used for the reference HPLC–UV method) were applied on CN-HPTLC surfaces and probed directly by FAPA-MS without further separation. The corresponding molecular maps are depicted in Fig. 4. As mentioned before, no analyte/matrix separation was performed to be able to investigate a potential matrix influence on the desorption/ionization and quantification efficiency of the analytes. The resulting concentrations determined by the isotope dilution method are summarized in Table 4. Similarly, to the data obtained for the samples with analyte/matrix separation, standard deviations were found to be minimal, supporting the fact that the desorption/ionization process is relatively stable with/without matrix present. For the calculation of the concentrations, the ROI was mainly used as stated in the materials and methods chapter.

**Table 4:** Caffeine concentrations in beverages and beverage extracts determined by direct FAPA-MS imaging of dried spots on CN-HPTLC plates. For energy drink and cola, 20 µL of standard and 80 µL of sample were mixed, and for coffee and tea, 25 µL of standard and 75 µL of sample were mixed. For the extract samples, 25 µL of standard and 75 µL of sample extract were mixed. A threefold determination for each beverage and beverage extract was performed

Beverage	Caffeine concentration (mg/mL)	RSD	Bias to reference^1^
Energy drink	0.321 ± 0.001^2^	≤ 1%^2^	− 1%^2^
	0.335 ± 0.001^3^	≤ 1%^3^	+ 3%^3^
Cola	0.104 ± 0.0001^2^	≤ 1‰^2^	+ 7%^2^
	0.095 ± 0.0002^3^	≤ 1%^3^	− 2%^3^
Coffee	0.785 ± 0.003^2^	≤ 1%^2^	+ 1%^2^
	0.767 ± 0.0005^3^	≤ 1‰^3^	− 1%^3^
Tea	0.284 ± 0.002^2^	≤ 1%^2^	− 17%^2^
	0.353 ± 0.0005^3^	≤ 1%^3^	+ 4%^3^

^1^Caffeine concentration by HPLC–UV: energy drink: 0.324 mg/mL, cola: 0.097 mg/mL, tea: 0.341 mg/mL, coffee: 0.778 mg/mL; RSD ≤ 2% for all HPLC–UV measurements

^2^Direct beverage analysis, no sample preparation

^3^Beverage extract after liquid–liquid extraction

It can be stated that the results based on chromatographic separation (Table 3) differ from those without a previous separation (Table 4). The concentration bias is higher for all beverages when a separation step on the plates is performed before sampling with the FAPA source. This is somewhat counterintuitive because one would expect that an analyte/matrix separation would help to reduce matrix effects (and potentially isobaric interferences). However, the separation did not improve the results. In addition, beverage and beverage extracts were compared and led to deterioration. As mentioned above, beverages and extracts were both investigated with the same method to better understand the matrix influence on the quantitative analysis. For the energy drink, cola, and coffee, the matrix did not seem to have a large influence on the results (bias to reference range from − 2 to 7%). In the case of tea, however, the calculated caffeine contents were found to better match the HPLC-UV results when a liquid–liquid extraction was used before direct CN-HPTLC-FAPA-MS analysis (− 17% without, + 4% with extraction step).

In addition to CN-HPTLC, RP-LiChrospher-HPTLC plates were investigated as well but the results will only be briefly discussed here. Beverages were probed with and without a preceding planar chromatography step. Sample extracts were probed directly by FAPA-MS. Results are summarized in Table 5. Similar to the CN-HPTLC experiments, direct quantitation values of caffeine in beverages deposited on RP-LiChrospher-HPTLC plates (without separation) are in good agreement with the reference values and feature low RSDs (≤ 3%). The largest concentration biases to reference values were found when tea was analyzed directly on the plate. Clearly, the desorption surface seems to have a significant influence on the results when one compares the different HPTLC materials. The most prominent differences were found with beverage extracts and with planar chromatography. Here, concentration biases of up to − 84% were found with RP-LiChrospher-HPTLC plates (Table 5) while biases with cyano-modified silica were smaller (Tables 3 and 4).

**Table 5:** Direct and quantitative caffeine analysis by FAPA-MS in different beverages and beverage extracts applied on RP-LiChrospher-HPTLC plates. The pure beverage was investigated with (bold) and without (italics) separation and the extract was investigated without separation (bold-italics)

Beverage	Caffeine concentration (mg/mL)	RSD	Bias to reference^1^
Energy drink	**0.303 ± 0.010 **^**2**^	**3% **^**2**^	** − 6% **^**2**^
	*0.319* ± *0.001 *^*3*^	≤ *1% *^*3*^	** − ***2% *^*3*^
	***0.548* ± *0.094 ***^***4***^	***17% ***^***4***^	** + *70% ***^***4***^
Cola	**0.041 ± 0.022 **^**2**^	**54% **^**2**^	** − 58% **^**2**^
	*0.090* ± *0.003 *^*3*^	*3% *^*3*^	** − ***7% *^*3*^
	***0.016* ± *0.005 ***^***4***^	***31% ***^***4***^	** − *84% ***^***4***^
Coffee	**0.888 ± 0.030 **^**2**^	**3% **^**2**^	** + 14% **^**2**^
	*0.825* ± *0.005 *^*3*^	≤ *1% *^*3*^	+ *6% *^*3*^
	***1.154* ± *0.143 ***^***4***^	***12% ***^***4***^	** + *48% ***^***4***^
Tea	**0.256 ± 0.022 **^**2**^	**9% **^**2**^	** − 25% **^**2**^
	*0.282* ± *0.006 *^*3*^	*2% *^*3*^	** − ***17% *^*3*^
	***0.445* ± *0.058 ***^***4***^	***13% ***^***4***^	** + *30% ***^***4***^

^1^ Caffeine concentration by HPLC–UV: energy drink: 0.324 mg/mL, cola: 0.097 mg/mL, tea: 0.341 mg/mL, coffee: 0.778 mg/mL; RSD ≤ 2% for all HPLC–UV measurements

^2^Caffeine in beverage after separation (bold)

^3^Caffeine in beverage without separation (italics)

^4^Caffeine in extract without separation (bold-talics)

### Quantification of caffeine by sample application on glass slides

**Fig. 5:**
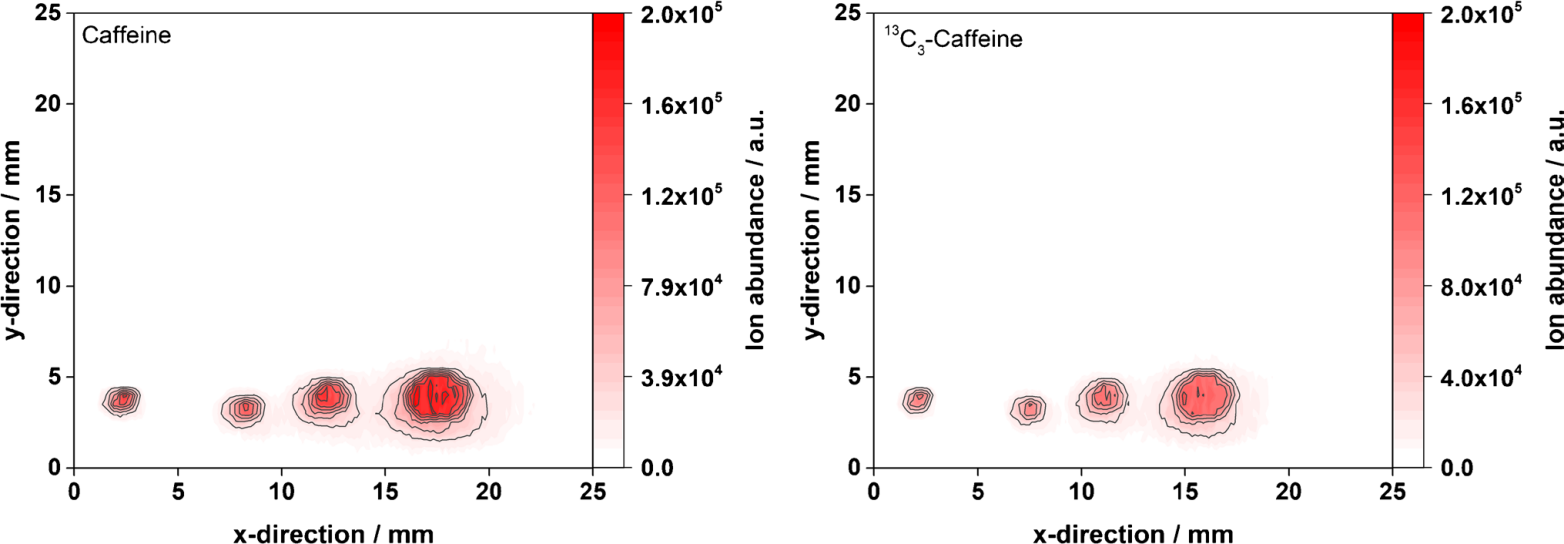
Mass spectrometric images of caffeine (left) and ^13^C_3_-caffeine (right) for the direct desorption/ionization of an energy drink from a microscope slide. FAPA source was operated at a helium flow rate of 750 mL/min and a discharge current of 35 mA. The deposited sample volume was 1 µL per spot (320 ng analyte/spot)

As alternative surfaces for dried droplet sampling, microscope glass slides and uncoated TLC glass plates were considered because they are readily available and very cheap compared to functionalized HPTLC plates. However, they would only be helpful if no matrix effects on the analyte and internal standard are expected, which would make planar chromatography advisable for analyte/matrix separation. It was expected from earlier work that spotting on glass would result in blurred and indistinct dried sample spots, especially when aqueous and polar-protic solvents were used. This was also observed here. An example of mass spectrometric images obtained after beverage analysis (energy drink) on a microscope slide is given in Fig. 5. Measurements on microscope slides and TLC glass for all other beverages were performed as well and results are provided as electronic supplementary material (ESM, Figs. S2–S11). As indicated in Fig. 5, spot shapes on microscope slides are heterogenous (*n* = 4, 320 ng/spot) compared to the spots obtained on coated HPTLC plates.

In addition, higher RSDs were observed for all beverages because the results were all based on real samples and no extraction or chromatography step was performed. Additionally, the sample application was not as reproducible as for the HPTLC plates. Nevertheless, the RSDs are still in an acceptable range (< 7%) for ADI-MS due to the use of an internal standard. Table 6 summarizes the calculated caffeine concentrations on the different glass surfaces. Caffeine quantitation in tea shows a consistent bias across the data yielding a lower concentration than the reference. Surprisingly, the bias is smaller compared to results with a preceding chromatographic separation (Table 3). When comparing the two glass surfaces, the microscope slides were found to be more reliable. For example, the determination of caffeine in cola on uncoated TLC glass resulted in a very large positive bias of over 500%.

**Table 6:** Calculated caffeine concentrations in different beverages applied on glass surfaces. For energy drink and cola, 20 µL of standard and 80 µL of sample were mixed, and for coffee and tea, 25 µL of standard and 75 µL of sample were mixed. For energy drink on microscope slides, a fourfold determination was performed. For the other measurements, a threefold determination was performed

Beverage	Caffeine concentration (mg/mL)	RSD	Bias to reference^1^
Energy drink	0.369 ± 0.016^2^	4%^2^	+ 14%^2^
	0.209 ± 0.014^3^	7%^3^	− 35%^3^
Cola	0.097 ± 0.002^2^	2%^2^	± 0%^2^
	0.623 ± 0.037^3^	6%^3^	+ 542%^3^
Coffee	0.827 ± 0.008^2^	1%^2^	+ 6%^2^
	0.813 ± 0.006^3^	≤ 1%^3^	+ 4%^3^
Tea	0.257 ± 0.001^2^	≤ 1%^2^	− 25%^2^
	0.287 ± 0.006^3^	2%^3^	− 16%^3^

^1^Caffeine concentration by HPLC–UV: energy drink: 0.324 mg/mL, cola: 0.097 mg/mL, tea: 0.341 mg/mL, coffee: 0.778 mg/mL, RSD ≤ 2% for all HPLC–UV measurements

^2^Values with regard to microscope slide

^3^Values with regard to TLC glass

Differences in the analyte signal response between microscope slides and uncoated TLC glass could be related to the different types of materials. The microscope slides consist of soda-lime glass, which is the most commonly used glass type. Information on the slides by the supplier (Carl Roth GmbH + Co. KG) revealed that the main chemical components are SiO2, Na2O, and CaO. No coatings or additives are used. Furthermore, the amount of MgO is reportedly relatively high with approximately 4% (to make the surface more hydrophilic). According to MACHEREY–NAGEL, the TLC glass material is thoroughly cleaned Pilkington Microwhite™. The main components reportedly are SiO2, Na2O, CaO, and MgO (approx. 4%). A significant difference between the two materials is the wettability of the surfaces. As stated before, it was difficult to reproducibly apply the liquid sample on the microscope slide due to its high hydrophilicity. Even small volumes of 1 µL lead to relatively broad spots. In contrast, liquid sample application on TLC glass did not show such a strong spreading. The effect was even more pronounced when methanolic extracts were applied. Here, the methanolic sample droplets were maintained on the surface without spreading and gave defined and reproducible spot shapes after drying on TLC glass slides.

### Transmission-mode FAPA-MS for caffeine quantification on stainless-steel meshes

**Fig. 6:**
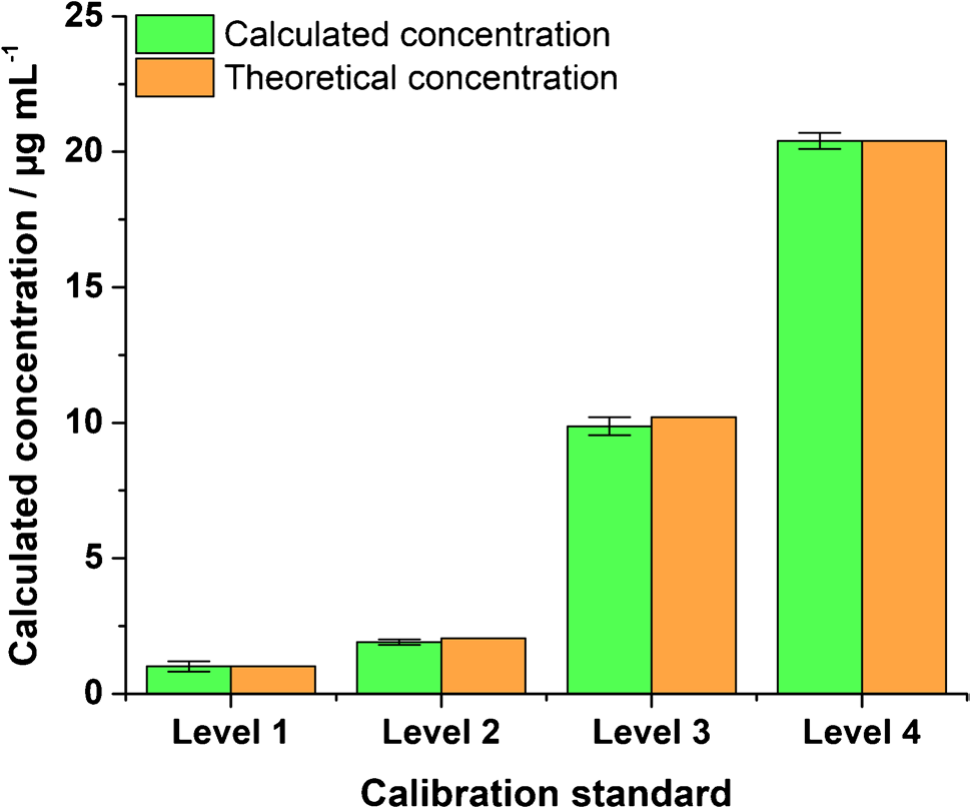
Comparison of theoretical caffeine concentrations of standard solutions and concentrations of respective standards measured by TM-FAPA-MS and calculated with the isotope dilution formula. Standard concentrations were 1.02 µg/mL (level 1), 2.04 µg/mL (level 2), 10.2 µg/mL (level 3), and 20.4 µg/mL (level 4)

As an alternative technique for analyte sampling in this study, a transmission-mode (TM) FAPA-MS approach was established using a steel mesh. This approach would be interesting when no spatial resolution or a preceding separation step would be required. First, the applicability of the transmission-mode approach was investigated by probing neat standards with known caffeine concentrations and without any matrix with the FAPA source. (cf. Figure 6). Quantification was performed as discussed above (cf. isotope dilution formula in Eq. S1). Due to the nature of the TM experiments, no molecular maps were generated and only ion intensities based on the chronograms were used for calculations. It was found that the results were quantitative (based on the isotope dilution formulae) and in excellent agreement with the expected values (in the 1–20 µg/mL range). Second, real beverages (with a significant matrix load) and beverage extracts were analyzed and the results are summarized in Table 7.

**Table 7:** TM-FAPA-MS analysis of caffeine in beverages and beverage extracts. Liquids were pipetted onto a steel mesh as follows: For energy drink and cola, 20 µL of standard and 80 µL of sample were mixed. For coffee and tea, 25 µL of standard and 75 µL of sample were mixed. For extract samples, 25 µL of standard and 75 µL of sample extract were mixed. A threefold determination for each beverage and beverage extract was performed

Beverage	Caffeine concentration (mg/mL)	RSD	Bias to reference^1^
Energy drink	0.333 ± 0.011^2^	3%^2^	+ 3%^2^
	0.243 ± 0.001^3^	≤ 1%^3^	− 25%^3^
Cola	0.104 ± 0.010^2^	10%^2^	+ 7%^2^
	0.074 ± 0.002^3^	3%^3^	− 24%^3^
Coffee	0.802 ± 0.034^2^	4%^2^	+ 3%^2^
	0.547 ± 0.003^3^	≤ 1%^3^	− 30%^3^
Tea	0.285 ± 0.004^2^	1%^2^	− 16%^2^
	0.240 ± 0.002^3^	≤ 1%^3^	− 30%^3^

^1^Caffeine content as determined by HPLC–UV: energy drink: 0.324 mg/mL, cola: 0.097 mg/mL, tea: 0.341 mg/mL, coffee: 0.778 mg/mL; RSD ≤ 2% for all HPLC–UV measurements

^2^Values with regard to real sample + internal standard

^3^Values with regard to extract + internal standard

The caffeine concentrations from direct measurements of the beverages (energy drink, cola, coffee) are surprisingly accurate when compared to the HPLC–UV (reference) results (+ 3–7% deviation). In beverage extracts, a negative bias on the order of 24–30% was observed (similarly to results with glass surfaces). Repeatability was found to be acceptable. Specifically, analysis of extracts resulted in slightly better RSDs (≤ 1–3%) compared to beverage results (RSDs 1–10%), which could be attributed to less matrix being present. A similar trend was also observed with other desorption surfaces as discussed above.

The main advantage of the transmission-mode approach is a simple and fast experimental procedure. After initial sample preparation, a small sample volume (in the order of 1 µL) is applied onto the mesh, the solvent is evaporated, and, finally, the mass spectrum is recorded, which takes about 15 s. However, it was found that the analyte signal of caffeine in a sample with matrix is significantly lower when compared to results with CN-HPTLC-FAPA-MS. It certainly depends on the type of application solvent, matrix load, and concomitants but for caffeine quantitation in beverages TM-FAPA-MS is considered less sensitive and less reliable at analyte concentrations below 1 ng of applied caffeine when compared to CN-HPTLC-FAPA-MS.

### Direct comparison of the ADI response of caffeine from different sampling surfaces

**Fig. 7:**
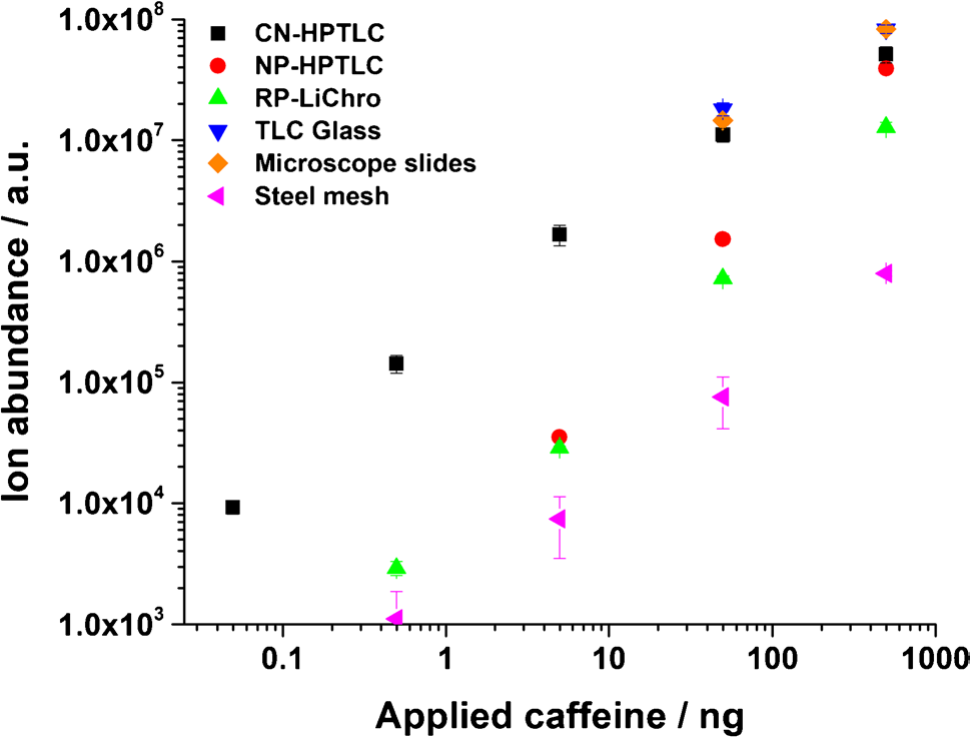
ADI response represented by the integrated ROI of caffeine as [M + H]^+^ versus the applied caffeine amount in ng ranging from 0.0498 to 498 ng on different surfaces. The data points are visually assigned to the respective surfaces, as illustrated in the upper left part of the figure. The FAPA source was operated at a helium flow rate of 750 mL/min and a discharge current of 35 mA. The deposited sample volume was 1 µL per spot as a methanolic solution including three spots per concentration step on each surface. Note the logarithmic scaling

One important fundamental question is whether or not the described phenomena can only be observed in real samples or also in pure analyte standards. As a direct comparatative study of the ADI response, different caffeine amounts were applied on the respective surfaces. In Fig. 7, the influence of the surface type on the analyte ion signal response is shown.

For pure caffeine, the two glass surfaces show the highest signal response (in the upper ng range) followed by CN-HPTLC and NP-HPTLC. A smaller analyte signal can be observed for RP-LiChrospher, and the response is even weaker for stainless-steel mesh. More interesting in this context is the probed total caffeine amount on the surface and the FAPA-MS sensitivity across different surfaces. Here, the main drawback of the glass surfaces comes into account. For the two highest applied amounts, all spots can easily be distinguished from the background, but at lower caffeine amounts, it is not possible to differentiate co-deposited spots spatially, which makes reproducible signal integration challenging. With the stainless-steel mesh, similar issues cannot occur because the spots are applied individually and spatially well separated from each other onto the mesh surface. However, as already mentioned, the response is significantly lower than for all other surfaces but good linearity in said range (498 pg–498 ng) can be achieved. The advantages of CN-HPTLC surfaces compared to all other studies surfaces can be seen throughout the data set. CN-HPTLC plates provide the best results over the total caffeine amounts studied (49.8 pg–498 ng). Because 49.8 pg of applied caffeine were reproducibly detectable (RSD < 12%), this was considered the lowest detectable concentration in this study. It is likely that lower values could be achieved, and a careful characterization of the instrumental and method LODs/LOQs is planned for a follow-up study. Additionally, CN-HPTLC plates guarantee a defined sample application to the surfaces, similar to the other two TLC surfaces. This is in accordance with the results on the beverage samples (see above). It should be noted, however, that the beverage samples are mostly aqueous solutions, whereas the caffeine standards were all applied as methanolic solutions. Potential solvent effects during the sample application may influence analyte diffusion on and into the surface. These, in turn, may then influence the desorption efficiency per given area. This potential solvent matrix effect will be investigated in the future in more detail.

## Conclusion

In this study, the influence of different sampling surfaces on the analyte signal response in FAPA-HR-MS was systematically investigated and accurate quantification of caffeine in different beverages was achieved based on an isotopically labeled standard. Different stationary phases were evaluated including NP-, RP-, and CN-HPTLC in addition to microscope slides and uncoated TLC glass. Also, results were compared to a stainless-steel mesh transmission-mode experiment. After careful optimization, caffeine was quantified successfully with CN-HPTLC-FAPA-HR-MS. The potential benefit of an analyte/matrix separation step before FAPA-HR-MS analysis was investigated but found to be not mandatory for this application, which is consistent with the basic idea of performing direct analysis with ADI-MS. The HPTLC surfaces served only as sample substrates when beverages were probed by FAPA-MS and no planar chromatography step was required to match HPLC–UV results. In addition, quantitation of caffeine in beverage extracts resulted in larger biases compared to direct beverage analysis in most cases. This finding may be explained by the different diffusional behavior of the respective samples which is highly influential for the desorption process and will be part of future studies on FAPA-MS including TLC surfaces as sample carriers. Across all investigated sample carriers, CN-HPTLC plates turned out to be the most efficient ones. Not only the analyte ion signals were significantly higher and RSDs were small but also all studied caffeine concentrations were detectable over a broad range. With respect to the polarity of the investigated surfaces, CN-HPTLC is considered medium-polar with cyanopropyl functionalities. RP- and NP-LiChrospher are non-polar and polar, respectively. However, the desorption/ionization efficiency cannot be explained based on differences in surface polarity alone. Properties such as surface roughness, homogeneity, and diffusion behavior should be considered for future studies as well. Because caffeine was used as the lead substance in aqueous or methanolic media in this study, no generalized conclusions for other compounds, solvents, and matrices should be made. Different substance classes and other surface chemistries should be investigated in the future to elucidate the influence of the surface on the desorption/ionization process in plasma-based ambient ionization. Overall, FAPA-MS combined with dedicated sample carrier surfaces has the potential to become an important method not only in rapid screening applications but also in quantitative analyses of complex samples.

## Supplementary Information

Below is the link to the electronic supplementary material.

Supplementary file1 (DOCX 2.30 mb)

## References

[1] Butt MS, Sultan MT (2011). Coffee and its consumption: benefits and risks. Crit Rev Food Sci Nutr.

[2] Bessada SMF, Alves RC, Oliveira M (2018). Caffeine-based food supplements and beverages: trends of consumption for performance purposes and safety concerns. Food Res Int.

[3] Fang WP, Meinhardt LW, Tan HW, Zhou L, Mischke S, Zhang DP (2014). Varietal identification of tea (Camellia sinensis) using nanofluidic array of single nucleotide polymorphism (SNP) markers. Hortic Res-England.

[4] Ashihara H, Sano H, Crozier A (2008). Caffeine and related purine alkaloids: biosynthesis, catabolism, function and genetic engineering. Phytochemistry.

[5] Ashihara H, Crozier A (2001). Caffeine: a well known but little mentioned compound in plant science. Trends Plant Sci.

[6] Perrone D, Donangelo CM, Farah A (2008). Fast simultaneous analysis of caffeine, trigonelline, nicotinic acid and sucrose in coffee by liquid chromatography-mass spectrometry. Food Chem.

[7] Bispo MS, Veloso MCC, Pinheiro HLC, De Oliveira RFS, Reis JON, De Andrade JB (2002). Simultaneous determination of caffeine, theobromine, and theophylline by high-performance liquid chromatography. J Chromatogr Sci.

[8] Buyuktuncel E (2010). Simultaneous determination of theobromine, paraxanthine, theophylline, and caffeine in urine by reversed-phase high-performance liquid chromatography with diode array UV detection. Anal Lett.

[9] Gallignani M, Torres M, Ayala C, Brunetto MD (2008). Determination of caffeine in coffee by means Fourier transform infrared spectrometry. Rev Tec Fac Ing Univ Zulia.

[10] Wang NY, Fu YC, Lim LT (2011). Feasibility study on chemometric discrimination of roasted arabica coffees by solvent extraction and Fourier transform infrared spectroscopy. J Agric Food Chem.

[11] Garrigues JM, Bouhsain Z, Garrigues S, de la Guardia M (2000). Fourier transform infrared determination of caffeine in roasted coffee samples. Fresenius J Anal Chem.

[12] Takats Z, Wiseman JM, Gologan B, Cooks RG (2004). Mass spectrometry sampling under ambient conditions with desorption electrospray ionization. Science.

[13] Cody RB, Laramee JA, Durst HD (2005). Versatile new ion source for the analysis of materials in open air under ambient conditions. Anal Chem.

[14] Shelley JT, Badal SP, Engelhard C, Hayen H (2018). Ambient desorption/ionization mass spectrometry: evolution from rapid qualitative screening to accurate quantification tool. Anal Bioanal Chem.

[15] Paglia G, Ifa DR, Wu CP, Corso G, Cooks RG (2010). Desorption electrospray ionization mass spectrometry analysis of lipids after two-dimensional high-performance thin-layer chromatography partial separation. Anal Chem.

[16] Pasilis SP, Kertesz V, Van Berkel GJ, Schulz M, Schorcht S (2008). HPTLC/DESI-MS imaging of tryptic protein digests separated in two dimensions. J Mass Spectrom.

[17] Van Berkel GJ, Ford MJ, Deibel MA (2005). Thin-layer chromatography and mass spectrometry coupled using desorption electrospray ionization. Anal Chem.

[18] Morlock G, Chernetsova ES (2012). Coupling of planar chromatography with direct analysis in real time mass spectrometry. Cent Eur J Chem.

[19] Kiguchi O, Oka K, Tamada M, Kobayashi T, Onodera J (2014). Thin-layer chromatography/direct analysis in real time time-of-flight mass spectrometry and isotope dilution to analyze organophosphorus insecticides in fatty foods. J Chromatogr A.

[20] Eichner F, Spangenberg B (2019). Optimized determination of caffeine, equol, and artemisinin by high-performance thin-layer chromatography-direct analysis in real time-time of flight-mass spectrometry. JPC-J Planar Chromatogr-Mod TLC.

[21] Gong XX, Zhang D, Embile IB, She Y, Shi SY, Gamez G (2020). Low-temperature plasma probe mass spectrometry for analytes separated on thin-layer chromatography plates: direct vs laser assisted desorption. J Am Soc Mass Spectrom.

[22] Winter GT, Wilhide JA, LaCourse WR (2017). Analysis of hop acids by thin-layer chromatography and the molecular ionization desorption analysis source (MIDAS) for mass spectrometry. Int J Mass Spectrom.

[23] Winter GT, Wilhide JA, LaCourse WR (2016). Molecular ionization-desorption analysis source (MIDAS) for mass spectrometry: thin-layer chromatography. J Am Soc Mass Spectrom.

[24] Kuhlmann C, Heide M, Engelhard C (2019). Fast screening and quantitative mass spectral imaging of thin-layer chromatography plates with flowing atmospheric-pressure afterglow high-resolution mass spectrometry. Anal Bioanal Chem.

[25] Ceglowski M, Smoluch M, Reszke E, Silberring J, Schroeder G (2016). Flowing atmospheric pressure afterglow combined with laser ablation for direct analysis of compounds separated by thin-layer chromatography. Anal Bioanal Chem.

[26] Shelley JT, Wiley JS, Hieftje GM (2011). Ultrasensitive ambient mass spectrometric analysis with a pin-to-capillary flowing atmospheric-pressure afterglow source. Anal Chem.

[27] Albert A, Shelley JT, Engelhard C (2014). Plasma-based ambient desorption/ionization mass spectrometry: state-of-the-art in qualitative and quantitative analysis. Anal Bioanal Chem.

[28] Gurdak E, Green FM, Rakowska PD, Seah MP, Salter TL, Gilmore IS (2014). VAMAS interlaboratory study for desorption electrospray ionization mass spectrometry (DESI MS) intensity repeatability and constancy. Anal Chem.

