# Supplementary material for: Quantitative detection of caffeine in beverages using flowing atmospheric-pressure afterglow (FAPA) ionization high-resolution mass spectrometry imaging and performance evaluation of different thin-layer chromatography plates as sample substrates
Source: Anal Bioanal Chem. 2022 Apr 20;414(15):4481–95. doi: 10.1007/s00216-022-04045-z (PMC9142459; doi:10.1007/s00216-022-04045-z)
Supplement: Supplementary file 1 — Supplementary file1 (DOCX 2.30 mb) [file 216_2022_4045_MOESM1_ESM.docx]

SUPPORTING INFORMATION

Quantitative detection of caffeine in beverages using flowing atmospheric-pressure afterglow (FAPA) ionization high-resolution mass spectrometry imaging and performance evaluation of different thin-layer chromatography plates as sample substrates

Maximilian Heide^†^, Cristian C. Escobar-Carranza^†^, Carsten Engelhard^†‡*^

^†^Department of Chemistry and Biology, University of Siegen, Adolf-Reichwein-Str. 2, 57076 Siegen, Germany

^‡^*Research Center of Micro- and Nanochemistry and (Bio)Technology,* University of Siegen, Adolf-Reichwein-Str. 2, 57076 Siegen, Germany

*Corresponding author: engelhard@chemie.uni-siegen.de

Table of Content

[1 Isotope dilution analysis for caffeine quantification S-2](#_Toc83829721)

[2 HPLC/UV method for validation of caffeine concentrations in beverages S-2](#_Toc83829722)

[3 Molecular maps on microscope slides and uncoated TLC glass S-3](#_Toc83829723)

# Isotope dilution analysis for caffeine quantification

Besides classic quantification based on external and internal standard calibration, which was performed for selected TM mode experiments, quantification using the isotope dilution analysis approach was performed according to

$\text{c}_{\text{Sample}}\text{=}\frac{\text{c}_{\text{Standard}}\text{∙}\text{V}_{\text{Standard}}\text{∙}\text{M}_{\text{Sample}}}{\text{V}_{\text{Sample}}\text{∙}\text{M}_{\text{Standard}}}\text{∙}\frac{\text{A}_{\text{Standard}}\text{-R}\left[ \frac{\text{12}_{\text{C}}}{\text{13}_{\text{C}}} \right]\text{∙}\text{B}_{\text{Standard}}}{\text{R}\left[ \frac{\text{12}_{\text{C}}}{\text{13}_{\text{C}}} \right]\text{∙}\text{B}_{\text{Sample}}\text{-}\text{B}_{\text{Standard}}}$ Equation (S1)

where *c_Sample_* is the unknown sample concentration, *c_Standard_* is the concentration of the labeled standard solution, e.g. ^13^C_3_-caffeine, *V_Standard_* and *V_Sample_* are the volumes of added sample solution and standard solution, *M_Standard_* and *M_Sample_* are the molecular masses of the respective isotopologues, *A_Standard_* and *A_Sample_* are the ^12^C-abundances in the respective isotopologues, *B_Standard_* and *B_Sample_* are the ^13^C-abundances in the respective isotopologues and R[^12^C/^13^C] is the measured ratio of the isotopologues. Most of the quantities are predefined by the sample and standard used or can easily be adjusted. The measured quantity R[^12^C/^13^C] is calculated by the measured intensities of the isotopologues. In the case of TM mode experiments it is simply the XICs intensity of caffeine divided by the XICs intensity of ^13^C_3_‑caffeine over the recording time of 12 s. For the molecular mapping experiments the respective integrated isotopologue spots obtained in the contour plots based on the summed up XICs defined as the region of interest (ROI) or the local maxima were used as the intensities to calculate the ratio R[^12^C/^13^C].

# HPLC/UV method for validation of caffeine concentrations in beverages

**External standard and beverage sample preparation.** For the HPLC/UV calibration approach a caffeine stock standard (c= 0.1 mg/mL) was prepared in methanol. Based on the stock standard a series of standards ranging from a caffeine concentration of 0.06 mg/mL to 0.75 mg/mL was diluted including a five calibration points.

The caffeine in the four beverages (Red Bull and Coca-Cola were purchased in local grocery markets, tea and coffee were self‑brewed) was extracted by liquid-liquid extraction. In a separatory funnel, the respective sample (5 mL) was mixed with bidistilled water (10 mL) and a Na_2_CO_3_ solution (20%, 1 mL). The aqueous phase was extracted with chloroform (3 x 20 mL) and the organic phases were combined. In the next step, the solvent was evaporated. After the solid residue was completely dried, the glass vaporizer flask was refilled with methanol (10 mL) and the solvent was evaporated. This step was repeated (5 mL and 3 mL of methanol) to ensure maximum recovery of the analyte. The solid residue was then dissolved in methanol (4 mL), transferred to a 5 mL volumetric flask and the flask was filled to the mark with methanol.

**Caffeine quantification in beverages with HPLC/UV.** An Agilent 1200 HPLC/UV system (Agilent Technologies, Santa Clara, CA, USA) was used for validation of the FAPA-TLC-MS results. Instrument control and data processing was performed with the Agilent OpenLab CDS Workstation 2.2 (Agilent Technologies, Santa Clara, CA, USA). For chromatographic separation an RP-18 column (XBridge, 4.6 mm x 75 mm, 2.5 μm particle size, Waters, Eschborn, Germany) was installed in the system. The mobile phase consisted of 45% methanol and 55% 0.1% aqueous formic acid. The flow rate was set to 0.5 mL/min for the isocratic run. An injection volume of 5 μL sample and standard was set and the injection syringe was rinsed with aqueous methanol (66%) after each injection. The detection wavelength was 273 nm and the total analysis time per injection was 4 min. The measurement sequence is shown in table S1.

**Table S1.** Measurement sequence for the HPLC/UV validation experiment.

| Blank → | Caffeine control → | Red Bull → | Coca-Cola → | Tea → | Coffee → |
| --- | --- | --- | --- | --- | --- |
| Blank → | Caffeine control → | Red Bull → | Coca-Cola → | Tea → | Coffee → |
| Blank → | Caffeine control → | Red Bull → | Coca-Cola → | Tea → | Coffee |

The external calibration graph for the analysis is shown in figure S1 which also includes the calibration formula and the coefficient of determination.





**Figure S1.** External calibration graph established with HPLC/UV for methanolic caffeine standard solutions ranging from 0.06 mg/mL to 0.75 mg/mL. Error bars are shown for n = 3 repetitive measurements each.

The obtained caffeine concentrations in the beverage extracts including their standard deviations are summarized in table S2.

**Table S2.** Resulting concentrations in the respective beverages obtained by HPLC/UV.

| **Beverage** | **Experimental concentration** |
| --- | --- |
| Red Bull | 0.324±0.006 mg/mL |
| Coca-Cola | 0.097±0.001 mg/mL |
| Tea | 0.341±0.004 mg/mL |
| Coffee | 0.778±0.012 mg/mL |

# Molecular maps on microscope slides and uncoated TLC glass

As mentioned in the study, microscope slides and uncoated TLC glass were used as additional sample carrying surfaces for direct spot-sampling which also omit chromatographic separation. The molecular maps are based on the mass ranges for caffeine and ^13^C_3_-caffeine listed in table 1 of the main manuscript. The molecular maps for cola, tea, and coffee as well as for all beverage extracts are depicted in Figures S2-S11.





**Figure S2.** Mass spectrometric images of caffeine (left) and ^13^C_3_-caffeine (right) on microscope slides for the cola sample. The FAPA source was operated at a helium flow rate of 750 mL/min and a discharge current of 35 mA. Deposited sample volume was 1 µL per spot (97 ng analyte/spot).





**Figure S3.** Mass spectrometric images of caffeine (left) and ^13^C_3_-caffeine (right) on microscope slides for the tea sample. The FAPA source was operated at a helium flow rate of 750 mL/min and a discharge current of 35 mA. Deposited sample volume was 1 µL per spot (341 ng analyte/spot).





**Figure S4.** Mass spectrometric images of caffeine (left) and ^13^C_3_-caffeine (right) on microscope slides for the coffee sample. The FAPA source was operated at a helium flow rate of 750 mL/min and a discharge current of 35 mA. Deposited sample volume was 1 µL per spot (778 ng analyte/spot).





**Figure S5.** Mass spectrometric images of caffeine (left) and ^13^C_3_-caffeine (right) on microscope slides for the energy drink extract. The FAPA source was operated at a helium flow rate of 750 mL/min and a discharge current of 35 mA. Deposited sample volume was 1 µL per spot (324 ng analyte/spot).





**Figure S6.** Mass spectrometric images of caffeine (left) and ^13^C_3_-caffeine (right) on microscope slides for the cola extract. The FAPA source was operated at a helium flow rate of 750 mL/min and a discharge current of 35 mA. Deposited sample volume was 1 µL per spot (97 ng analyte/spot).





**Figure S7.** Mass spectrometric images of caffeine (left) and ^13^C_3_-caffeine (right) on microscope slides for the tea extract. The FAPA source was operated at a helium flow rate of 750 mL/min and a discharge current of 35 mA. Deposited sample volume was 1 µL per spot (341 ng analyte/spot).





**Figure S8.** Mass spectrometric images of caffeine (left) and ^13^C_3_-caffeine (right) on microscope slides for the coffee extract. The FAPA source was operated at a helium flow rate of 750 mL/min and a discharge current of 35 mA. Deposited sample volume was 1 µL per spot (778 ng analyte/spot).





**Figure S9.** Mass spectrometric images of caffeine (left) and ^13^C_3_-caffeine (right) on uncoated TLC glass for the energy drink (lower row of spots) and cola (upper row of spots) sample. The FAPA source was operated at a helium flow rate of 750 mL/min and a discharge current of 35 mA. Deposited sample volume was 1 µL per spot (324 ng/spot for energy drink and 97 ng analyte/spot for cola).





**Figure S10.** Mass spectrometric images of caffeine (left) and ^13^C_3_-caffeine (right) on uncoated TLC glass for the coffee (lower row of spots) and tea (upper row of spots) sample. The FAPA source was operated at a helium flow rate of 750 mL/min and a discharge current of 35 mA. Deposited sample volume was 1 µL per spot (778 ng/spot for coffee and 341 ng analyte/spot for tea).





**Figure S11.** Mass spectrometric images of caffeine (left) and ^13^C_3_-caffeine (right) on uncoated TLC glass for the energy drink extract (top and first row of spots), cola extract (second row of spots), tea extract (third row of spots), and coffee extract (lowest and fourth row of spots). The FAPA source was operated at a helium flow rate of 750 mL/min and a discharge current of 35 mA. Deposited sample volume was 1 µL per spot (324 ng analyte/spot for energy drink, 97 ng/spot for cola, 341 ng analyte/spot for tea, and 778 ng/spot for coffee).
